# Progesterone signaling in cardio-renal physiology and pathophysiology

**DOI:** 10.3389/fendo.2026.1837458

**Published:** 2026-07-03

**Authors:** Jenny Nguyen, Alison J. Kriegel

**Affiliations:** Department of Physiology, Medical College of Georgia, Augusta University, Augusta, GA, United States

**Keywords:** cardiorenal, heart, kidney, progesterone, vasculature

## Abstract

Progesterone is a multifunctional steroid hormone essential for reproductive function, yet its roles in cardiovascular and renal physiology remain comparatively less understood than those of estrogen. While its classical actions in regulating the menstrual cycle, promoting endometrial receptivity, and maintaining pregnancy are well-established, progesterone also signals through both genomic pathways via nuclear receptors, and non-genomic mechanisms via membrane receptors. These receptors are expressed outside of reproductive tissues, including the vasculature, heart, and kidney, supporting broader systemic effects of progesterone that are not yet fully defined. When compared to the current understanding of estrogen-mediated regulation in these tissues, the mechanisms conferring progesterone-mediated regulation of cardiovascular and renal function are less well studied. This review explores the contemporary understanding of progesterone signaling on cardiorenal physiology and pathophysiology in females and males, highlighting the need for enhanced methodological reporting in studies in this field and further investigation into the integrative roles of progesterone signaling.

## Highlights

Progesterone is increasingly recognized as an important regulator of cardiovascular and renal function, extending beyond its established roles in reproduction. There is a continued and growing interest in understanding the mechanisms underlying cardiorenal physiology and pathophysiology in women (155, 220, 221). Despite the emerging insights discussed here, the cardiorenal effects of progesterone remain less characterized than those of other sex steroids. Additionally, there is a clear benefit to the research community by enhancing transparency and rigor in the reporting of study methods and results in this area, as well as efforts to standardize research approaches (222, 223). Key mechanisms of action, including receptor-specific actions, tissue-specific signaling pathways, signaling pathway modifiers, and sex-dependent differences in progesterone signaling, have not been studied extensively and represent major areas for further research in both men and women.

## Introduction

Progesterone is a pleiotropic steroid hormone that plays a central role in coordinating reproductive, cardiovascular, and renal physiology. Primarily recognized for its essential function in the menstrual cycle, implantation, and maintenance of pregnancy (1–4), progesterone also exerts widespread systemic effects through genomic and non-genomic signaling pathways (3, 5). In the cardiovascular system it modulates endothelial function (6, 7), vascular tone, and autonomic balance, thereby contributing to blood pressure regulation (8). Progesterone has numerous effects on the heart including cardiac excitability (9, 10) and, cardiomyocyte contraction (11), and others. Progesterone also impacts renal physiology through effects on sodium handling (12, 13), renal hemodynamics and interactions with the renin-angiotensin-aldosterone (RAAS) system (14). These integrated actions of progesterone facilitate adaptive functions in cardiovascular and renal function to maintain homeostasis during changes in reproductive status.

Many studies aimed at understanding the actions of female sex steroids have focused on the effects of estrogen, combined estrogen and progesterone, or synthetic progestins (15, 16). Here we review studies that elucidate progesterone-specific effects on cardiorenal physiology in animal models and clinical studies. Additionally, we summarize published methods of progesterone delivery in rodent models and the circulating levels measured following these changes. Collectively, the diverse and integrated actions highlighted underscore the essential role of progesterone in reproductive function and adaptive changes in cardiorenal physiology.

## Progesterone synthesis and degradation

**Figure 1:**
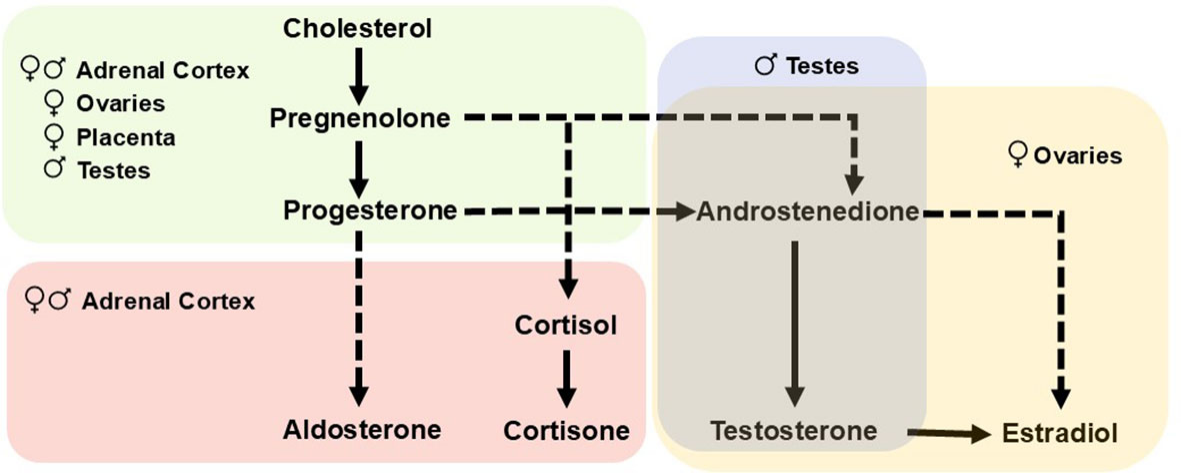
Summary of steroid hormone synthesis. This simplified diagram outlines the steroid hormone synthesis pathway with primary sites of production in humans outlined by tissue site and sex. Note that rodents do not produce cortisol. Dashed lines indicate multi-step processes.

Progesterone is synthesized in the ovary, testes, and the adrenal cortex (**Figure 1**). Progesterone is produced by the corpus luteum early in pregnancy, followed by continued production by the placenta (1). In recent years, it has also been recognized that other cell types can synthesize progesterone (17). Ovarian production of progesterone is stimulated with the release of follicle-stimulating hormone (FSH) and luteinizing hormone (LH) by the pituitary gland (2).

Progesterone and other steroid hormones are synthesized from cholesterol (reviewed extensively elsewhere (5, 18, 19), and briefly outlined in **Figure 1**). Cholesterol, utilized for steroid hormone synthesis, is largely obtained from receptor-bound lipoproteins that are internalized by endocytosis (20, 21) and ultimately trafficked to the mitochondria (19). The first step in steroid hormone synthesis occurs within the mitochondria, where the side chain of cholesterol is cleaved by the cytochrome P450 side-chain cleavage enzyme (CYP11A1) to form pregnenolone (22). Pregnenolone is then released and converted to progesterone by 3β-hydroxysteroid dehydrogenase/Δ^5^-Δ^4^ isomerase (3βHSD). Pregnenolone and progesterone serve as precursors for the production of mineralocorticoids, glucocorticoids, testosterone, and estradiol through a cascade of tissue-specific enzymatic reactions (2, 5) (**Figure 1**). Chronic potassium depletion can induce progesterone production by the adrenal glands (23, 24). Various types of stress can also increase progesterone production by adrenal glands (25–32).

Circulating progesterone is predominantly carried in the blood bound to proteins, exerting its biological functions in an endocrine manner (5). Circulating progesterone has a short half-life of approximately 5 minutes (3).

## Progesterone receptors

**Figure 2:**
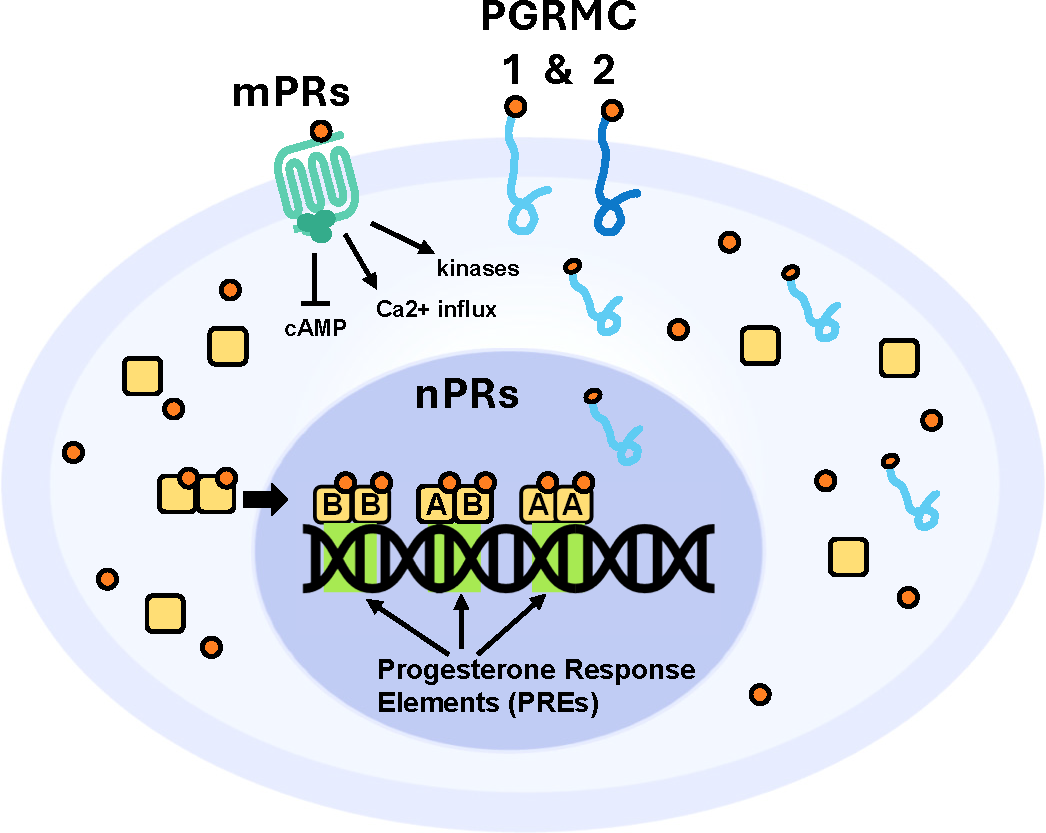
Cellular localization and signaling mechanisms of major progesterone receptors. Nuclear progesterone receptors (nPRs; PR-A and PR-B) are ligand-activated transcription factors that, upon binding progesterone, dimerize and regulate gene expression by interacting with progesterone response elements (PREs) and transcriptional cofactors. nPR signaling regulates gene expression relating to proliferation, differentiation, and tissue remodeling. Membrane progesterone receptors (mPRs; PAQRs) are plasma membrane-associated receptors that mediate rapid, primarily non-genomic signaling through second messenger signaling including inhibition of cAMP, activation of MAPK/ERK and PI3K/AKT signaling, and regulation of intracellular calcium, initiating various cell responses. Progesterone receptor mediated components (PGRMC1/2) are membrane-associated adaptor proteins localized to plasma membranes, intracellular organelles and the nucleus. PGRMCs do not act as classical receptors, rather they act as adaptors or scaffolding proteins. PGRMC1 has been more extensively researched than PGRMC2, and has roles in processes including cell survival, proliferation, migration, and stress responses.

Progesterone has three main receptor subtypes: nuclear progesterone receptors (nPRs), membrane progesterone receptors (mPRs), and progesterone receptor membrane components (PGRMC) (**Figure 2**). Each mediate different signaling pathways. It is important to note that progesterone can also bind to mineralocorticoid receptors (MR) (33, 34), where it antagonizes the effects of aldosterone (34–39), as well as androgen receptors (40). Synthetic progestins can have significant off-target effects on mineralocorticoid, glucocorticoid, and androgen receptors (41).

### Nuclear progesterone receptors

A single gene encodes three different protein isoforms of nPRs (PR-A, PR-B, and PR-C), which are produced by post-translational cleavage. These receptors mediate canonical progesterone signaling through DNA-binding and regulation of gene expression. Rapid non-genomic signaling was reported to occur through nPR activation of Src kinase (42). Additionally, a mitochondrial progesterone receptor (PR-M) with non-nuclear function, has also been reported to be transcribed from the common gene (43).

Unbound nPRs within the cytoplasm complex with chaperone proteins. Progesterone binding changes nPR conformation, resulting in dissociation from chaperone proteins and homo- or heterodimerization of PR-A and PR-B. The nPR dimer binds progesterone response elements (PREs) in the promoter region of progesterone target genes, initiating the formation of a complex of coactivators or corepressors and transcriptional machinery (44). The composition of nPR isoforms within the dimer determines whether the progesterone signal activates or inhibits translation of the target gene in a cell- and condition- specific manner.

PR-B is the full-length receptor and contains an N-terminus region important for transcriptional activity and activation (45), while PR-A is a cleaved isoform of PR-B that lacks the N-terminus region. PR-A can also directly inhibit PR-B actions (46). Some studies indicate that many promotors are regulated by both PR-A and PR-B in a similar manner (47). Additionally, PR-A and PR-B abundances are similar in human tissues targeted by progesterone (48). Post-translational modification can also reversibly alter the activity of PR-B (49). Other studies show that PR-A can inhibit both PR-B and estrogen receptor signaling (50). Loss of PR-A impairs normal progesterone response in ovarian and uterine tissues, but not the mammary gland. Loss of PR-B has the opposite effect (51). PR-C is a further truncated nPR isoform, understood to be produced by the uterus during labor (52). PR-C lacks the DNA-binding domain seen in PR-A and PR-B but retains the region needed for progesterone binding (53). It is understood to regulate PR-A or PR-B transcriptional activity by forming heterodimers (52, 54).

nPRs are found in ovaries (55, 56), uterine tissue (57, 58), fallopian tubes (58, 59), placenta (60), testes (61), brain (62), pancreas (63), bone (64), urinary tract (65), and kidneys (66). The transcript expression of nPR was similarly expressed in male and female nephrons, but only in the connecting tubule, cortical collecting duct, and outer medullary collecting duct, and not earlier segments (66).

### Membrane progesterone receptors

Membrane progesterone receptors (mPRs) mediate rapid transmembrane progesterone signaling. They consist of three main isoforms that belong to the progestin and adipoQ (PAQR) family: mPRα (PAQR7), mPRβ or (PAQR8), and mPRγ (PAQR5). These 7-transmembrane domain proteins bind progesterone with high affinity (67, 68) and act in both reproductive and non-reproductive tissues (69, 70). Extracellular progesterone binds to mPRs, activating G proteins (Gi/o) and subsequently leading to reduced cAMP, activation of the MAPK pathway, and changes in intracellular calcium levels. mPRα is expressed in many tissues and has important roles in numerous processes ranging from stimulation of sperm motility (68, 71) and oocyte maturation (67, 72–74) to inhibition of apoptosis (75, 76) and neuronal GnRH release (77). mPRα and mPRβ can mediate progesterone transactivation of PR-B in human myometrium (78). Understnding of mPRγ function is limited, but it is widely expressed across tissues and play similar roles in oocyte maturation and other processes (79–82).

### Progesterone receptor membrane component 1/2

Progesterone receptor membrane components 1/2 (PGRMC1 & PGRMC2) are single transdomain proteins that participate in some progesterone signaling, but are not typical receptors (47). PGRMC isoforms contain cytochrome b5-like heme-binding domain (83); however, the cellular expression and function differ between receptors (84).

PGRMC1 is understood to mediate cell proliferation and is highly expressed in uterine tissue during the proliferative phase (85–87) and in various cancers. PGRMC1 has been found in endoplasmic reticulum, Golgi apparatus, inner acrosomal membrane, and the nucleus (88–91), in addition to the cell membrane, suggesting diverse actions within the cell.

PGRMC1 mediates progesterone signaling through protein-protein interactions with P450 proteins (92), epidermal growth factor receptor (EGFR) (93), plasminogen activator inhibitor RNA-binding protein-1 (PAI-RBP1) (94, 95) (96, 97), and has effects on PI3K/Akt signaling (98), mitochondrial function, metabolism, and apoptosis (99, 100). The nuclear localization of monomeric PGRMC1 is likely related to its apparent function in transcriptional regulation, as this is the mechanism by which it inhibits apoptosis (100, 101) or cell cycle regulation (102, 103).

Within the heart, PGRMC1 is known to regulate metabolism (104, 105), while little is known about its function in the kidney. The transcript encoding PGRMC1 is expressed throughout nephron segments in male and female mice (106). Expression was highest in proximal tubules, connecting tubules, cortical collecting ducts and outer medullary collecting ducts, and lower in the ascending limbs (66).

Comparatively less is known about PGRMC2; however, its general cellular localization is similar to PGRMC1 (107). Several studies suggest that PGRMC2 has a role in preventing cellular proliferation and is induced by progesterone (108), resulting in increased expression in the uterus during the secretory phase (109). Loss of PGRMC2 increases the proliferation and migration rate in ovarian cancer cells (107). Additionally, PGRMC2 plays an important role in intracellular heme transport (110).

## Progesterone metabolism

The majority of progesterone metabolism occurs in the corpus luteum of the ovaries and the liver, and resulting metabolites are excreted into the urine (111). As mentioned above, progesterone antagonizes aldosterone signaling through MRs. One would anticipate that progesterone would reduce sodium retention, but in conditions of high circulating progesterone levels (such as the luteal phase and in pregnancy), progesterone is metabolized to deoxycorticosterone, a MR agonist, which causes sodium and water retention by the kidney (5, 112). The kidney can metabolize progesterone to metabolites (*e.g*. 17α-hydroxyprogesterone and 20α-dihydroprogesterone), which are much less potent aldosterone antagonists (39, 112–114), as well as testosterone and dihydrotestosterone, which can transactivate the androgen receptor (40, 112).

## Progesterone effects on reproductive physiology

Progesterone is considered a female sex hormone associated with pregnancy and the effects of progesterone are best understood in the context of female reproductive physiology. In female puberty, progesterone is primarily known for its role, alongside estrogen, in maturing developing breasts to create lactating competent mammary glands. Most bioactive progesterone is produced by the ovaries as an important regulator of the menstrual cycle from puberty to menopause.

Progesterone promotes epithelial growth in the mammary gland, contributing to alveologenesis (115). Progesterone is essential for the proliferation of the mammary ductal epithelium and lobular-alveolar system required for lactation (116). High progesterone levels during pregnancy inhibit prolactin secretion, allowing the development of a lactation-competent gland without lactation (117–119).

In humans, the menstrual cycle is a monthly cycle of hormonal changes that prepares the body for a potential pregnancy by altering the ovaries and endometrium in parallel (120). It is separated into distinct phases: the menstrual phase, the follicular phase, ovulation, and the luteal phase. The follicular phase begins at the onset of menstruation, when estrogen and progesterone hormones levels are low. Ovulation is the release of an egg from a mature follicle for potential fertilization, and this is triggered by a cascade of hormones from the hypothalamus and pituitary gland (121). The endometrium undergoes a proliferative phase during the follicular phase of the menstrual cycle, followed by a secretory phase during the luteal phase (122). Estrogen and progesterone, produced from the corpus luteum, are important facilitators of the proliferative and secretory phase of the endometrial cycle. Estrogen levels increase during the proliferative phase, leading to rapid growth of the endometrial lining, whereas progesterone predominates during the secretory phase to halt the thickening and prepare for blastocyst blaimplantation (119, 122, 123).

In the secretory phase, the rise in progesterone induces a cellular transformation of the endometrial stromal cells (ESCs) in a process called decidualization (4, 124). In the absence of an implanted fertilized oocyte, the cyclical drop in progesterone levels at the end of the luteal phase will trigger menstrual shedding of the decidualized ESCs (deESCs) (124). But if implantation of a fertilized oocyte does occur, the ovarian corpus luteum continues to produce estrogen and progesterone in response to human chorionic gonadotropin (hCG) (1), and the properties of the transformed deESCs help provide a nutritive environment essential for embryo implantation and placental development. While estrogen is known for its role in mediating endometrial proliferation (125), progesterone is important for stimulating endometrial angiogenesis and vascularization in early pregnancy (4, 122, 126, 127).

Decidualized ESCs (DeESCs) display many phenotypic changes, including changes to morphology, structural properties, and molecular properties. DeESCs become more epithelioid in morphology, with increased nuclear size and expansion of the endoplasmic reticulum and Golgi complex (128). Metabolic changes include a large increase in mitochondrial network and cellular accumulation of lipids and glycogen (124, 128, 129), as well as increased expression of antioxidant proteins (130). Cytoskeletal properties change (131), and cells undergo mesenchymal-to-epithelial transition (132) marked by an increase in E-cadherin and cytokeratin and a decrease in vimentin proteins (130, 132, 133). DeESCs also exhibit increased expression of extracellular matrix proteins, including fibronectin, laminin, and collagen type IV during. Additional gap junctions and adherens junctions also form to increase intercellular communication (134–136). DeESCs can secrete numerous proteins, including prolactin and insulin-like growth factor binding protein-1 (IGFBP-1) (137), as well as CCL2 (138) and CXCL12 (139), which indirectly contribute to maternal immune tolerance in pregnancy (140, 141).

Progesterone mediates increased production of pro-angiogenic vascular endothelial growth factor (VEGF) and angiopoietin by deESCs, which together play a critical role regulating vascular permeability, blood vessel formation, proliferation, migration, and differentiation in the endometrium (126, 127, 142). Mature capillaries penetrate the granulosa layer of cells, resulting in a significant increase in vascularization and blood flow in the endometrium (2). As pregnancy progresses beyond 6 weeks, the contribution of estrogen and progesterone from the corpus luteum declines, and production by the developing placenta increases (1). The placenta converts large amounts of maternal and fetal cholesterol to pregnenolone and progesterone (143).

## Cardiorenal effects of progesterone monotherapy in women

As in many animal studies, human studies of progesterone primarily examine its effects as an adjunct to estrogen replacement (144–148). In the section below, we will explore the reported effects of limited studies on progesterone monotherapy in women. Although the therapeutic effects of synthetic progestins have been explored in several studies (149), we focus here on progesterone.

In hormonally intact women, progesterone treatment increased GFR, uric acid clearance, and kallikrein excretion. Progesterone treatment also augmented urinary excretion of sodium and progesterone (150). In another study conducted in premenopausal females, hormone levels were first blocked with a gonadotropin-releasing hormone (GnRH) antagonist prior to progesterone replacement (151). Subjects were then observed for their acute response to an isotonic saline load. In this study, women receiving progesterone had lower plasma atrial natriuretic peptide concentrations and higher plasma aldosterone concentrations, without changes in fluid handling or sodium excretion in response to an acute saline load (151). Investigators acknowledged that progesterone replacement induced a relatively low plasma progesterone concentration, and noted that a higher level of progesterone may have different effects on fluid or sodium regulation (151).

Studies on the effects of progesterone monotherapy in older adults have also been performed (152, 153). Progesterone treatment reduced blood pressure in older men and women (152). A three-month study of postmenopausal women at low cardiovascular risk found no adverse or beneficial effect on blood pressure or other factors influencing their Cardiovascular Risk Profile (153). It remains unclear if women at higher cardiovascular risk would benefit from progesterone monotherapy.

## Insight on progesterone effects in cardiorenal physiology from female animal models

Sex differences in cardiorenal physiology, as well as alterations in females after menopause, are well-established and have been extensively discussed (154–159). In the remaining sections of this review, we discuss the cardiorenal effects of progesterone revealed through *vivo* and *ex vivo* mammalian studies and cell models in contexts outside of pregnancy. Only those studies including experimental groups treated with progesterone alone are summarized, and the reported treatment conditions and resulting circulating progesterone levels from *in vivo* rat studies are summarized in **Table 1**.

**Table 1:** Conditions of chronic progesterone (P) delivery in rat studies.

Study by Author	Rat strain	Sex	Age or weight	Status	Hormone washout period	Progesterone treatment conditions				Circulating [P] (ng/ml or *pg/ml)		
						Vehicle	P Dosage	Route	Duration	Intact	Ovx	+P
LaMarca, et al. (144)	SD	F	15 wks	Ovx	none	pellet	(21d) 200 mg	s.c	14d	--	37±6	75±9
Rattanasopa, et al. (152)	SD	F	8-9 wks	Ovx	2 d	corn oil	1mg/rat, 3x/wk	s.c.	10 wks	20.8±1.1	6.4±1.7	12.6±0.9
Goldstein, et al. (154)	SD	F	8-9 wks	Ovx	none	pellet	(21d) 50 mg	s.c.	17d	--	--	--
Gambling, et al. (168)	W	F	12 wks	Ovx	2 wks	corn oil	3.3 mg/kg once	s.c.	24 hrs	--	--	--
Herak-Kramberger et al. (172)	W	F+M	8 wks	Ovx/ Gnx	none	sunflower oil	2.5 mg/kg per d	s.c.	6 wks	--	--	--
Cheema et al. (175)	W	F	225 ± 20g	Ovx	none	sesame oil	10 mg/kg per d	s.c.	14 d	15.7 ± 5.7	2.0 ± 0.3	22.7 ± 2.9
Rojas-Vega et al. (176)	W	F	12 wks	Ovx	1 mo	10% ethanol/ olive oil	20 mg/kg per d	i.p.	3 wks	--	11 ± 3	91 ± 6
Graceli et al. (178)	W	F	3 mo	Ovx	7 d	corn oil	1.7 mg/kg per d	s.c	7d	17.8 ± 2.8	4.1 ± 0.9	16.9 ± 1.7
Gohar, et al. (180)	SD	F	13-14 wks	Ovx	none	pellet	(21d) 25 mg	s.c.	18d	92.2 ± 5.5	42.9 ± 3.9	42.4 ± 8.4
Montezano et al. (181)	W	F	6 wks	Ovx	none	pellet	(21d) 250 mg	--	7 wks	*515 ± 4	*246 ± 12	*565 ± 19
Al-Trad et al. (185)	W	F	55-60d	Ovx	--	sesame oil	10 mg/kg per 2 d	s.c.	10 wks	25.9 ± 0.6	13.2 ± 3.3	32.1 ± 3.9
Ghasemi et al. (186)	W	F	178.4 ± 1.4g	Ovx	5d	sesame oil	2-25mg/kg per 5 d	i.m.	20 d	--	--	--
Abramicheva et al. (187)	W	F	12 wks	Ovx	none	Propylene glycol	10 mg/kg per day	i.p.	2 wks	--	--	--
Sabolic, et al. (193)	W	F+M	10-12 wks	Gnx	8 d	sunflower oil	2.5 mg/kg per d	s.c.	8d	--	--	--
Ljubojevic, et al. (194)	W	F+M	20-25d	Gnx	6 wks	sunflower oil	2.5 mg/kg per d	s.c.	14d	--	--	--
Ljubojevic, et al. (195)	W	F+M	10-12 wks	Gnx	8 d	sunflower oil	2.5 mg/kg per d	s.c	8d	--	--	--
Sandhi et al. (196)	W	M	200-250g	Intact	n/a	olive oil	10 mg/kg once	i.p	25 hrs	1.46 ± 0.09	--	4.66 ± 0.19

Rat strains are listed for Wistar (W) and Sprague Dawley (SD) and sex is female (F) and/or male (m). Age or weight is shown at the beginning of the study. Hormonal status variables tested in the context of P treatment include ovariectomy (Ovx), gonadectomy (Gnx) or hormonally intact (Intact). The hormone washout period indicates the time between Ovx or Gnx and P treatment. Hormone delivery routes include intraperitoneal (i.p.), intramuscular (i.m.) and subcutaneous (s.c.). Data not included/reported indicated as “--“. .

## Cardiovascular effects of progesterone

### Progesterone regulation of the vasculature

There is limited understanding of the effects of progesterone on cardiovascular function compared to those of estrogen. Progesterone lowers vascular resistance through two mechanisms: increased endothelial nitric oxide (NO) production and direct effects on smooth muscle. Progesterone stimulates endothelial nitric oxide synthase (eNOS) expression (6) and activity via mPRα^7^, increasing NO production and mediating relaxation of surrounding vascular smooth muscle cells via mPRs (160). Progesterone also has direct effects, reducing contraction and promoting calcium influx in coronary smooth muscle cells (161). Progesterone signaling through PGRMC1 contributes to sustained vasodilation by inhibiting nitric oxide degradation and reducing oxidative stress (93). Progesterone has also been shown to antagonize the antioxidant-mediated protective effect of estrogen on the vasculature, and progesterone treatment of cultured vascular smooth muscle cells increased the production of reactive oxygen species (ROS) (162). Finally, research in ovariectomized rhesus monkeys suggests that progesterone treatment can also attenuate coronary vasospasm, possibly by reducing expression of thromboxane A2 receptors (TxA_2_) (163). TxA_2_ receptors promote vasoconstriction through various mechanisms (164).

Despite the reported vasodilatory effects of progesterone, chronic progesterone replacement in ovariectomized adult female Sprague Dawley rats did not alter mean arterial blood pressure (acute) or change the contractile response of the carotid artery to phenylephrine, acetylcholine, or sodium nitroprusside (165). By contrast, progesterone replenishment in ovariectomized ewes reduced resting MAP and increased plasma volume, when compared to ovariectomized or hormonally intact ewes (8). The reduction in MAP was accompanied by a reduction in angiotensin II in response to hypotension (8). A separate study in rats demonstrated that the reduced pressor response to angiotensin II observed in pregnancy is unlikely to be mediated by either progesterone or estrogen (166).

Studies on progesterone effects in atherosclerotic models suggest that it provides no protective benefit during the development of associated pathology (167, 168). In female rabbits fed an atherogenic diet, ovariectomy increased atherosclerosis and collagen synthesis in the aortic arch, and this was not improved by progesterone treatment (167). A similar lack of progesterone benefit on atherosclerosis was observed in cynomolgus monkeys (168).

### Progesterone regulation of cardiac function

Studies that have focused on the effects of progesterone on cardiac function indicate that it regulates diverse processes including cardiac excitability (9, 10), cardiomyocyte contraction (11), metabolism (169, 170), oxidative stress (169) and protein synthesis (171), as well as mediating acute protection from myocardial damage in cardiac ischemia-reperfusion injury (172). Details of those studies are outlined here and in **Table 1**.

Progesterone clearly affects cardiac excitability, though the mechanisms remain unclear. Females have higher heart rates and longer heart rate-corrected QT intervals than males after puberty (173–175), and women with long QT syndrome are at a higher risk of developing a type of ventricular tachycardia called Torsades de Pointes (TdP) (176–178) with various drugs. The effect of estrogen and progesterone has been studied in TdP (10). When ovariectomized rabbits were treated with progesterone, the incidence of TdP induced by dofetilide, an inhibitor of the delayed rectifier potassium current, was reduced in isolated, perfused hearts (9). In another study in this rabbit model, progesterone treatment reduced the prolongation of the ventricular action potential duration induced by quinidine (10), a sodium and potassium channel blocker that reduces cardiac excitability (179).

An antiarrhythmic effect of progesterone has also been noted. Bisphenol A (BPA), an estrogenic endocrine disrupter, has been shown to have acute proarrhythmic actions and therefore alter myocyte calcium handling in female rat cardiomyocytes; progesterone counterbalances the proarrhythmic alteration through mediated-initiated signaling involving G_i_ protein and Phosphoinositide 3-kinases (PI3Ks) (180). A similar anti-arrhythmic effect was seen in rabbits with impaired cardiac repolarization when treated with progesterone (181). In coronary smooth muscle cells, both progesterone and testosterone may inhibit calcium entry through non-voltage gated channels or potentially suppress other contractile mechanisms (161).

Studies in mice indicate that progesterone reduces the rate of cardiomyocyte contraction in females by reducing cardiomyocyte sensitivity without altering the calcium transients (11). Feridooni et al. reported that progesterone attenuates and slows contractions in hearts from female cells only; however, there was no effect on cellular mechanisms that regulate calcium release from the sarcoplasmic reticulum (11). The authors concluded that the slowed contraction is not due to changes in cellular calcium homeostasis (11).

There have been reports of progesterone effects on cardiomyocyte growth processes including maturation (182), pregnancy induced physiological cardiac hypertrophy (183), and cardiac muscle protein synthesis (171). Progesterone supplementation promoted proliferation in cardiomyocytes from postnatal day 7 mice, as indicated by increased immunostaining of Ki-67, PH3, and Aurora B CMs (184). In addition to promoting proliferation, it was also noted that progesterone treatment improved cardiac function in adult mice after myocardial infarction via ligation of the left anterior descending coronary artery (184).

Studies on cardiac metabolism suggest that progesterone has several modifying effects. Acute perfusion of isolated hearts from ovariectomized female rats reduces lipoprotein lipase (LPL) mRNA expression compared with vehicle-perfused controls (185). Lipoprotein lipase is important for fatty acid uptake from the circulation (186). Loss of ovarian hormones with ovariectomy has also been associated with impairment in cardiac mitochondrial function (187). Progesterone treatment in ovariectomized rats did not correct reductions in ATP production, but it did reduce the stimulated production of reactive oxygen species (ROS) (169). Progesterone treatment also significantly reduced the number of mitochondria per cardiomyocyte area compared with either ovariectomized or hormonally intact controls (169).

Cardiomyocyte protein synthesis was found to be stimulated by progesterone treatment in ovariectomized rats and blocked by mifepristone, a nuclear progesterone receptor and glucocorticoid receptor antagonist (171). In this study, an increase in plasma volume was also reported with progesterone treatment, compared with ovariectomy alone, and this was not prevented by mifepristone treatment (171).

Progesterone delivery to hormonally intact female rats reduced the area of infarcted cardiac tissue resulting from left descending artery occlusion (172). Progesterone treatment also reduced inflammatory markers, improved free radical scavenging, and enhanced contractile function (172). In this study, progesterone was introduced at minute 30 of a 60-minute occlusion, and data were collected after 60 minutes of reperfusion, suggesting that this was an acute effect (172).

## Renal effects of progesterone

Though sex differences in renal reabsorption have been largely attributed to androgen effects of enhancing tubular reabsorption of sodium and water, a growing number of studies have focused on the effects of estrogen and progesterone in these processes. Many studies indicate that female sex hormones regulate urine osmolarity through endocrine effects on osmoreceptors and vasopressin (15), or through sex differences in gene expression and renal function (188). The majority of this work has focused on the effects of estrogen signaling (15) and progesterone replacement in ovariectomized animals, which is provided in combination with estrogen replacement (16, 189). Progesterone has been historically viewed as renoprotective in various models of kidney injury, but there are many gaps in how this may be mediated. In this section, we discuss studies that examine the effects of progesterone replacement in ovariectomized animal models of kidney disease to isolate the progesterone-specific effects on the kidney.

### Water reabsorption

The expression of AQP1, which is vasopressin-insensitive and constitutively expressed in the kidney (190, 191), was moderately upregulated by progesterone treatment in the renal cortex and outer stripe of ovariectomized rats (192). The aquaporin 2 (AQP2) channel, expressed in the principal cells of the collecting duct, is highly sensitive to vasopressin (193), and has been shown to be increased in expression in pregnant rats (194). Progesterone replacement in ovariectomized females had little to no effect on urine output, osmolarity, or AQP2 (195). The results of this study suggest that progesterone does not stimulate AQP2 upregulation (195); however, the circulating progesterone levels achieved with hormone replacement were lower than those reported in pregnant rats (195).

### Ion transport

The effect of progesterone signaling on nephron ion transport regulation has also been explored. In female rats the activity of Na^+^-Cl^-^ cotransporter (NCC), expressed in the early distal convoluted tubule, was increased by progesterone (196). The renal expression of plasma membrane Na^+^/Ca^2+^ exchanger (NCX) family member NCKX3, also expressed in the distal convoluted tubule, is higher in female mice (197). Progesterone delivery in ovariectomized females or male mice did not alter NCKX3 expression (197). The transcript expression of ENaC subunits -α and -β was unchanged 24-hours after progesterone treatment in adult ovariectomized female rats, but ENaC-γ was upregulated (16). Another study reported that progesterone treatment restored a reduction in renal Na^+^/K^+^-ATPase activity, not expression, in ovariectomized rats (198).

A study of isolated proximal and distal tubules from male and female rabbits was designed to determine the effects of progesterone on apical transport mechanisms (12). Proximal tubules incubated with progesterone exhibited no change in apical transport of Ca^2+^ or Na^+^, but distal tubule cells exhibited a rapid increase in Ca^2+^ and a decrease in Na^+^ transport under the same treatment conditions (12). Interestingly, progesterone treatment at a very low concentration inhibited Ca^2+^ uptake, while higher concentrations enhanced the Ca^2+12^. Though this study supports the effects of progesterone signaling independent of nPR, it included both males and females, and the sex distribution within each experiment was not described.

### Autonomic function in the kidney

The effects of progesterone on mechanisms impacting renal function beyond the nephron have also been explored. Ovariectomy in female rats was found to increase catecholamine concentrations in both the plasma and kidney tissue, compared to hormonally intact controls (198). Progesterone replacement in ovariectomized rats restored plasma catecholamine concentrations to control levels and greatly attenuated renal catecholamine concentrations (198). These studies included group controls that underwent renal denervation, allowing them to conclude that progesterone may attenuate catecholamine release by renal nerves (198). Additionally, they found that progesterone replacement failed to attenuate the increased fractional excretion of sodium (FE_Na+_) regardless of renal innervation (198).

### Progesterone regulation of the renal vasculature

Expression of transcripts encoding receptors ET-A and ET-B for the vasodilatory peptide endothelin -1 (ET-1) was reduced in the renal cortex and increased in the inner medulla of ovariectomized female rats (199). Progesterone replacement did not significantly alter these changes in expression (199). Progesterone also had no effect on renal preproendothelin expression in ovariectomized female rats (165).

### Progesterone effects on renal pathology

Several studies have also aimed to evaluate the effect of progesterone in various models of renal pathology. Progesterone replacement was determined to confer renal protection, independently or in combination with estrogen replacement, in the DOCA-salt model of hypertension, when initiated at the time of Ovx (200). While estrogen and combined estrogen and progesterone treatment attenuated blood pressure in this model, replacement of progesterone alone did not (200). This suggests that progesterone-mediated renal protection in this model may have occurred through pressure-independent mechanisms. The authors speculate that this protection may relate to its reported actions of inhibiting the proliferation of glomerular mesangial cells or antagonizing effects on the mineralocorticoid receptor (200–202). An early study of the effect of progesterone on DOCA salt hypertension in males reported that progesterone increased sodium excretion and reduced blood pressure, suggesting this occurred by antagonizing the mineralocorticoid signaling (13).

In the streptozotocin (STZ) model of diabetic nephropathy, progesterone administration to ovariectomized female rats suppressed the progression of kidney damage as indexed by a reported reduction in urinary albumin to creatinine ratio, increased mRNA expression of podocin and mRNA expression of transforming growth factor beta-1 (TGFβ1), fibronectin and angiotensin II Type 1 (AT1) receptors (203) Progesterone treatment in ovariectomized female rats subjected to the cisplatin model of AKI suggested that progesterone dosage may importantly modulate the protective benefit of progesterone on markers of oxidative stress in a dose-dependent manner (204).

In 2023, Abramicheva and colleagues published a study in which they performed unilateral ureteral obstruction in female rats, with and without ovariectomy (205). Some ovariectomized females were also treated with progesterone. The transcript levels of various fibrosis markers were elevated in all UUO groups, including those with ovariectomy and progesterone treatment (205). In the context of UUO, neither the presence of endogenous ovarian hormones nor supplemental progesterone conferred protection against the upregulation of various fibrosis markers in kidney tissue. The mRNA expression levels of PAQR5 revealed that ovariectomy and UUO both reduced PAQR5 mRNA in levels in kidney tissue, while only those with both ovariectomy and UUO exhibited reduced PGR expression (205). They conclude that the reduced PAQR5 expression is associated with renal fibrosis and may reduce renal sensitivity to protective progesterone signaling (205). It is important to note that fibrosis is a severe model of kidney injury, but it is not the only pathological change that occurs during the progression of UUO. Plasma progesterone levels were not reported in this study (**Table 1**).

## Renal effects of progesterone in males

The effects of progesterone on male physiology have been explored, particularly in the context of progesterone loss with aging (206). It is important to note that progesterone receptors are expressed in many male tissues. nPRs are expressed particularly in the prostate, epididymis, testis, and male mammary gland (207). There is growing evidence that membrane progesterone receptors are widely expressed in both males and females. mPRs are expressed in the brain, sperm membranes, and gastrointestinal tract, among other tissues (70, 208–210) and their roles in these tissues are not well understood.

Several rodent studies have reported effects of progesterone treatment on tubular transporter expression in male rats (211–213). Studies in the kidney show that female rats exhibit higher expression of the proximal tubule (S3) sodium-glucose transporter SGLT1 (Scl5a1) than males (211). Compared to hormonally intact male controls, castrated rats show increased SGLT1 expression in the renal cortex and outer stripe, but low-dose progesterone delivery had no additional effect on SGLT1 expression (211). Progesterone delivery to castrated male rats had no effect on SGLT1 expression in the renal cortex or outer stripe, but castration alone (211). Similarly, the expression of proximal tubule (S3) organic anion transporter (OAT) OAT2 was higher in hormonally intact adult female rats than males or ovariectomized females, and progesterone treatment of castrated males did not augment OAT2 expression (212). By contrast, OAT1 expression was increased by progesterone treatment in castrated males (213). In 2015, Sandhi et al. reported that delivery of progesterone to male rats prior to 40 minutes of bilateral renal ischemia improved creatinine clearance and other indices of renal function measured 24 hours following reperfusion. Additionally, several indices of oxidative stress and inflammation, including lipid peroxidation, S-acetyl-glutathione (SAG), glutathione (GSH), myeloperoxidase (MPO), and catalase activity, were also improved by progesterone treatment prior to renal ischemia-reperfusion in male rats (214).

## Gaps in progesterone research models

It is important to note that both the method of progesterone delivery, dosages, treatment durations, and resulting plasma concentrations differ among the studies reviewed here (**Table 1**). Enhanced reporting of details of both methodological approaches and the efficacy of hormone replacement is likely to improve reproducibility in experimental models and clearly resolve the cardiorenal effects of progesterone. For example, several studies reported the use of mifepristone as a progesterone receptor antagonist (214, 215), without acknowledging its powerful dual effect as an inhibitor of the glucocorticoid receptor at higher dosages (216). Mifepristone is used to control Cushing’s Disease-associated hyperglycemia in patients without surgical treatment options (217, 218). The potential effect of mifepristone on the glucocorticoid receptor should be evaluated in studies where it is used to antagonize progesterone to avoid confounding the interpretation of assumed progesterone actions in various disease models.

There is also a need for research models that can isolate cell- and tissue-specific effects of progesterone signaling relevant to cardiorenal function and pathology. Inducible genetic editing of different progesterone receptors in specific cell types within the vasculature, heart, and kidneys, would help to isolate the functional effect of progesterone within various organs without significant developmental and reproductive impairment (116, 219).

## Limitations

The goal of this review was to provide the research community with a focused discussion of studies in which progesterone-specific effects on cardiorenal physiology could be isolated from those of estrogen. Subsequently, a limitation of this review is that the effects of progesterone in combination with estrogen are not explored.
